# Identification of Key Microorganisms and Metabolic Pathways Associated with the Formation of Off-Flavour Compounds in the Pit Mud of Strong-Flavour Baijiu

**DOI:** 10.3390/foods15152731

**Published:** 2026-08-03

**Authors:** Xiaofeng Zhang, Linjia Sun, Liu Yang, Xuesi Li, Zhenhua Cao, Chunmei Pan

**Affiliations:** 1 College of Life Sciences, Henan University of Animal Husbandry and Economy, Zhengzhou 450046, China; 2 College of Biological Engineering, Henan University of Technology, Zhengzhou 450001, China; 3 School of Life and Health Sciences, Hubei University of Technology, Wuhan 430068, China; 4 Henan Songhe Wine Industry Co., Ltd., Zhoukou 477262, China

**Keywords:** pit mud, microbial community, metabolic pathways, off-flavour compounds

## Abstract

Off-flavours represent one of the most prevalent and severe causes of deteriorating pit mud quality in Strong-Flavour Baijiu production. However, the key compounds responsible for these off-flavours and their formation mechanisms remain poorly understood, thereby limiting quality control in the production process of Strong-Flavour Baijiu pit mud. To investigate the origin of off-flavours in pit mud, this study employed gas chromatography–mass spectrometry coupled with metagenomic methods to compare samples from normal and off-flavoured pit mud. The results show that the main cause of off-flavours in pit mud is the abnormal accumulation of acids such as heptanoic acid and hexanoic acid due to imbalanced nutrient ratios, along with insufficient synthesis of key esters including ethyl hexanoate, ethyl butyrate, and ethyl lactate; microorganisms such as *Fermentimonas*, *Methanoculleus*, *Hortaea* and *Proteiniphilum*, which are non-strictly anaerobic and acid-sensitive in the pit mud, are key microbes associated with these odour compounds. Furthermore, based on the annotation from the KEGG database, this study further inferred the possible microbial metabolic pathways that could lead to the formation of these off-flavour compounds, including starch and cellulose degradation pathways, biosynthesis pathways of valine, leucine, and isoleucine, pyruvate metabolism pathways, and butyric acid metabolism pathways. In summary, the study systematically analysed the key substances related to the odour of the pit mud of strong-flavour Chinese Baijiu and its microbial sources, providing a theoretical basis for the quality regulation of the pit mud during production and developing high-quality artificial pit mud.

## 1. Introduction

As one of the traditional aroma types of Chinese baijiu, Strong-Flavour Baijiu is highly favoured by consumers for its rich pit aroma, mellow flavour, and smooth, sweet, and refreshing finish [1]. The production of Strong-Flavour Baijiu employs multiple grains as raw materials, utilises medium-to-high-temperature daqu as the fermentation agent, and employs pits as fermentation vessels for solid-state fermentation. The walls and base of these pits are lined with pit mud, with pit fermentation being a defining characteristic of the Strong-Flavour Baijiu brewing process [2]. Pit mud harbours diverse functional microorganisms. These microbes catalyse esterification pathways to generate various volatile flavour compounds. These volatile compounds are transferred to the fermenting mash through material exchange and subsequently enter the distillate during distillation, becoming integral components of the liquor’s flavour profile [3]. Consequently, the quality of pit mud is paramount to the quality of the baijiu produced.

However, during the succession of microorganisms in pit mud, factors such as changes in the nutrient elements of the pit mud, interactions between microbial communities, and production process operations may lead to microbial imbalance (e.g., excessive proliferation of aerobic bacteria inhibiting the growth of caproate-producing bacteria). This imbalance can cause issues such as an imbalance in volatile metabolic components, resulting in unpleasant off-flavours like a ‘muddy stench’ in the pit mud and ultimately leading to a significant decline in its quality [4]. 3-Methylindole and 4-methylphenol have been proven to be the key compounds that contribute to the formation of the ‘muddy odour’ of the pit mud. Excessive accumulation of organic acids such as butyric acid and caproic acid, as well as their interaction with 4-methylphenol, may cause the odour of the pit mud [5]. However, these studies have primarily focused on describing the key compounds responsible for the off-flavour perception in pit mud. The mechanisms governing the production and formation of these off-flavour compounds remain unexplained, making it difficult to effectively control their generation. The presence of off-flavour compounds is intrinsically linked to microbial metabolism during baijiu fermentation. For instance, the formation of odorous substances such as 3-methylindole, 4-methylphenol, and p-cresol in pit mud is primarily associated with microbial amino acid metabolism and lignin degradation processes [4]. Metagenomic sequencing data reveal significant correlations between the concentrations of these compounds and the abundance of specific microbial genera, including *Clostridium*, *Methanospirillaceae*, and *Desulfovibrio* [6]. Therefore, investigating the potential relationship between microbial communities and odorous compounds in off-flavour fermentation may help elucidate the formation patterns of these compounds, thereby guiding the regulation of microbial communities and enhancing the flavour characteristics of baijiu.

This study examined two types of fermentation pits, normal pit mud and off-flavoured pit mud, to identify the underlying compounds responsible for the odour and its microbial production. To this end, sensory evaluation was combined with headspace solid-phase microextraction coupled with gas chromatography–mass spectrometry (HS-SPME-GC-MS) to identify the sensory characteristics and off-flavour compounds of both pit muds. Metagenomic sequencing was employed to investigate the microbial communities within the two pit muds. Furthermore, relationships between off-flavour compounds and key microorganisms were revealed, and potential metabolic pathways for key off-flavour compounds were elucidated. This study provides insights into the off-flavour compounds and their generation within pit mud of Strong-Flavour Baijiu, offering guidance for enhancing baijiu quality.

## 2. Materials and Methods

### 2.1. Sample Collection

**Figure 1:**
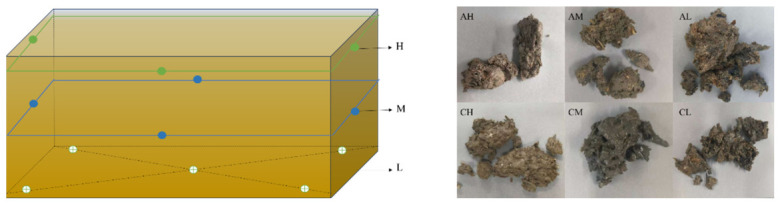
Sampling locations and samples of pit mud.

Samples were collected from fermentation pits at Songhe Liquor Co., Ltd. in Zhoukou City, Henan Province, China. Two wine vats of different quality but the same age were selected from the production workshop: those with discoloured, foul-smelling, coarse-textured mud exhibiting off-flavours such as rancidity, raw earth, or putrefaction were designated as off-flavour pits. Three samples were randomly selected from each quality cellar as parallel samples (n = 3). Each cellar was divided into the upper layer, middle layer and lower layer according to its spatial position. The stratified sampling method is shown in Figure 1. Three random samples from the upper and middle layers served as parallel replicates. Employing a five-point sampling method, 100 g of sample was collected from each sampling point (Figure 1). High-quality pits were designated as pit C, while off-flavoured pits were labelled as pit A. Samples were sealed in sterile bags, with one portion stored at −80 °C for DNA extraction and another portion kept at 4 °C for other analyses.

### 2.2. Determination of Volatile Compounds

This method follows the approach of Gao Jiangjing et al. [7], using a semi-quantitative analysis method. Take 2 g of pit mud, add 4 mL of 60% ethanol-saturated saline solution, and incorporate 3 µL of internal standard (n-pentyl acetate, 2-methylhexanoic acid, tert-amyl alcohol). Vortex for 5 min, then sonicate in an ice bath for 5 min. After allowing the mixture to settle and separate, the organic upper layer was collected. This extraction was repeated three times, and all upper layers were combined. The combined extract was nitrogen-evaporated to a volume of 250 µL.

Gas chromatography conditions: TG-WAXMS column (30 m × 0.25 mm, 0.25 µm, 1420N26088P); carrier gas: high-purity helium (99.999%), flow rate 1.00 mL/min; injection port temperature 250 °C; split injection, split ratio 30:1; solution delay 2 min; temperature programme: initial temperature 30 °C held for 2 min, ramped at 3 °C/min to 100 °C, then at 15 °C/min to 220 °C, held for 8 min.

Mass spectrometry conditions: Ionisation energy 70 eV; transfer line temperature 230 °C; ion source temperature 230 °C; monitoring mode: multi-reaction monitoring (MRM), scan time 300 ms; quadrupole temperature 150 °C; solution delay 5 min.

### 2.3. Genomic DNA Extraction and Metagenomic Sequencing

Quality-filtered reads for further analysis were obtained by processing raw sequencing reads. Concurrently, sequencing adapters were removed from the reads using Cutadapt (v1.2.1). Subsequently, low-quality reads were trimmed using the sliding window algorithm in fastp (v0.23.2). BMTagger (v1.1) was then employed to align reads against host genomes, eliminating host contamination. (after optimisation, we obtained 784018266 microbial sequences, as shown in Supplementary Material Figure S1). Following quality-filtered read acquisition, Kraken2 (v2.0.8) performed taxonomic classification of metagenomic sequencing reads from the pit sludge samples against the RefSeq database (encompassing genomes of bacteria, fungi, viruses, archaea, and microbial eukaryotes). Concurrently, each sample was assembled using Megahit (v1.1.2) with meta-large preset parameters. Clustering analysis was performed using MMseqs2 (v3.0) in ‘easy-linclust’ mode, setting the sequence similarity threshold to 0.95 and the coverage threshold for shorter contigs to 90% for clustering generated contigs (length > 300 bp). In ‘taxonomy’ mode, MMseqs2 aligned redundant contigs against the NCBI-nt database to determine the lowest common ancestor taxon. Genes within contigs were predicted using MetaGeneMark (v3.38). Concurrently, coding sequences across all samples were clustered via MMseqs2. Gene abundance was assessed using Salmon (v1.10.1) in combination with the counts-per-million method. MMseqs2 annotates gene function in ‘search’ mode by comparing non-redundant genes against the Kyoto Encyclopedia of Genes and Genomes (KEGG), the Evolutionary Gene Ontology: Unsupervised Homology Clusters (EggNOG), and the Carbohydrate Active Enzymes (CAZy) databases. EggNOG and Gene Ontology (GO) annotations were obtained using EggNOG-mapper (v2.1.3), whilst GO slim and KEGG ortholog (KO) annotations were acquired via map2slim (v0.15). (www.metacpan.org) and the KO-based annotation system, respectively.

### 2.4. Nutrient Element Detection

Moisture content determination: electric heating drying method; pH measurement: direct pH meter determination; ammonium nitrogen: indophenol blue colorimetric method; available phosphorus: ammonium fluoride–hydrochloric acid colorimetric method; available potassium: sodium tetraphenylborate turbidimetric method; organic matter: potassium dichromate titration method; calcium content: EDTA titration method; total acidity: acid–base neutralisation titration method.

### 2.5. Data Analysis

Nutrient element and α-diversity indices were examined using one-way analysis of variance (ANOVA) in SPSS Statistics 2022, with *p* < 0.05 deemed statistically significant. Data were plotted and statistically analysed using Excel 2021 and Origin 2021 software, with results expressed as mean ± standard deviation. The mass spectra of volatile flavour compounds were compared and identified with the NIST 17 database, and their relative contents were processed and analysed using SPSS Statistics 2022 and Excel 2021.The relative abundance plots of dominant species at the genus level, LEfSe multi-level species hierarchy trees, and LDA discriminant scatter plots were generated using the Meiji platform. The Spearman correlation coefficient (ρ) and significance (P) between dominant microorganisms (relative abundance > 1%) and volatile compounds were calculated using the ‘hmisc’ package in R. Symbiotic networks were constructed based on |ρ| > 0.6 and *p* < 0.05, then visualised in Cytoscape software (v.3.60). Principal Component Analysis (PCA) and partial least squares (PLS) analyses were performed using SIMCA software (v.1.0.1, Umetrics AB, Umea, Sweden). Additional statistical analyses and graph generation were conducted in Origin 2021 and R (v3.4.3). *p*-values < 0.05 were considered statistically significant.

## 3. Results and Discussion

### 3.1. Screening of Key Compounds for Off-Flavours of Pit Mud

**Figure 2:**
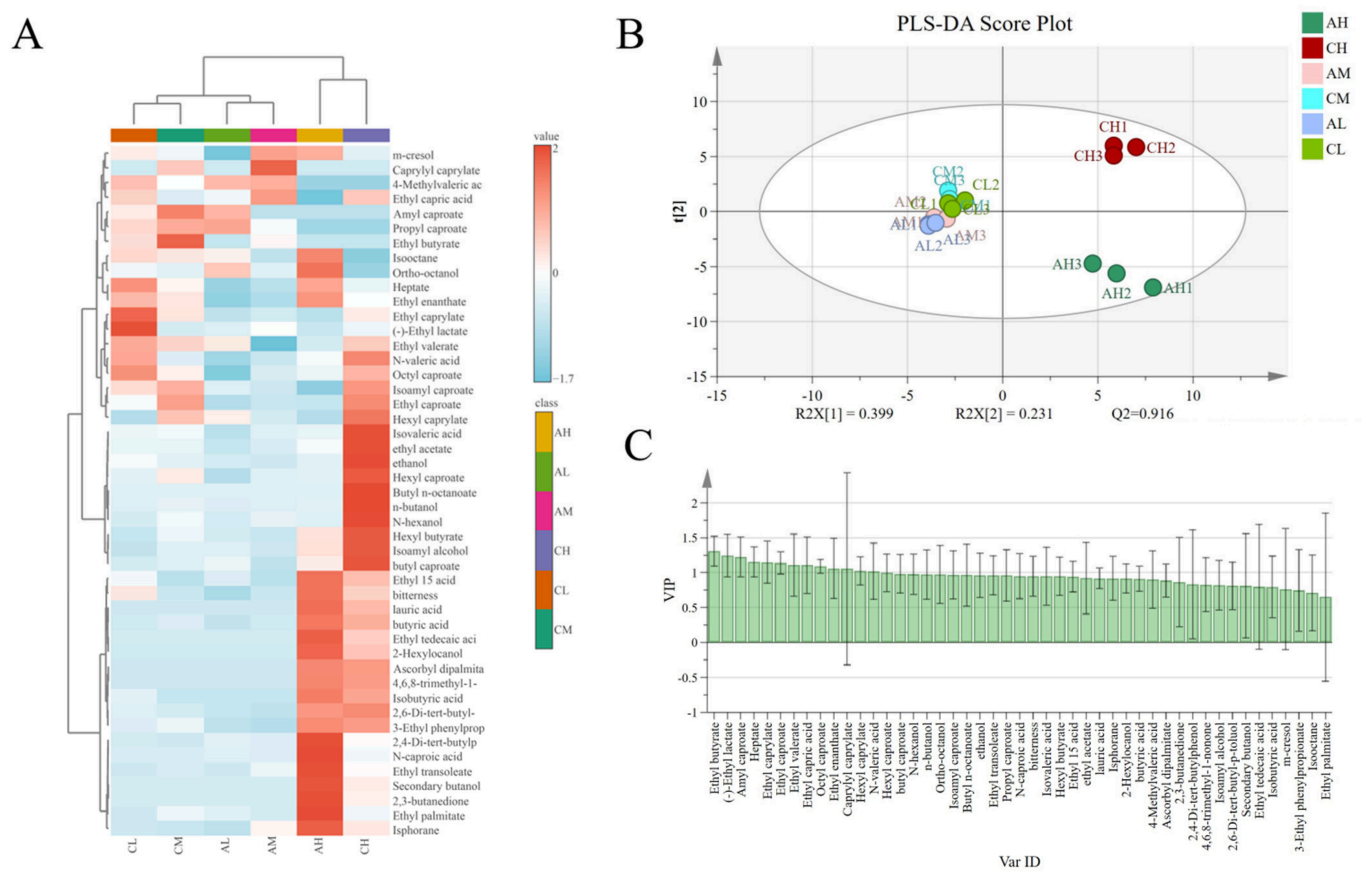
Differential analysis of volatile metabolites in sludge from normal and off-flavoured pit mud ((**A**): Volatile metabolites of normal and off-flavor pit mud; (**B**): PLS-DA of Volatile Metabolites in normal and off-Flavor pit mud; (**C**): Key volatile metabolites of normal and off-Flavor pit mud).

To identify the key compounds responsible for the off-flavours in pit A’s fermentation, we analysed the volatile compounds in both high- and low-quality pit mud. The results showed that a total of 47 volatile compounds were detected in the pit mud, including 24 esters, six alcohols, 10 acids, three aldehydes and ketones, three phenols, and 11 other compounds, as shown in Figure 2A. The significant difference analysis indicated that the average content of 4-methylvaleric acid in pit A was 11.57 mg/kg, significantly higher than 8.92 mg/kg in pit C (*p* < 0.005); heptanoic acid had an average content of 32.54 mg/kg in pit A, significantly higher than 21.36 mg/kg in pit C (*p* < 0.005); heptate had an average content of 346.83 mg/kg in pit A, significantly higher than 246.19 mg/kg in pit C (*p* < 0.005). These compounds have unpleasant odours and their abnormal accumulation may be the main source of the pit mud’s sour and musty smell. In contrast, the content of various characteristic aroma compounds in pit C was significantly higher than that in pit A, including ethyl butyrate in pit C with an average content of 18.56 mg/kg, significantly higher than 4.21 mg/kg in pit A (*p* < 0.005); (-)-lactic acid ethyl ester in pit C had an average content of 105.68 mg/kg, significantly higher than 30.12 mg/kg in pit A (*p* < 0.005); ethyl hexanoate in pit C had an average content of 434.76 mg/kg, significantly higher than 98.87 mg/kg in pit A (*p* < 0.005). These ester substances are the core contributors to the soft aroma of the pit mud. To further reveal the differences in volatile substances between pit A and pit C, partial least squares discriminant analysis (PLS-DA) was used to analyse the volatile compounds of the pit mud samples. The R^2^Y and Q^2^ values of the rank-sum test based on the PLS-DA model were 0.974 and 0.707, respectively, indicating that the model has good accuracy and predictability (Figure 2B). The results showed that there were significant differences in various compounds between the different levels of different pits, and a total of 14 key flavour substances (VIP > 1) were identified, mainly including 12 esters, such as octanoic acid ethyl ester and decanoic acid ethyl ester, and two acids, namely heptanoic acid and octanoic acid (Figure 2C). Among them, the contents of heptanoic acid (32.54 mg/kg), heptanoic acid ethyl ester (15.34 mg/kg), and octanoic acid (3682.93 mg/kg) were significantly higher in pit A, causing the pit mud to have a strong sour and musty smell, while the core aroma esters such as ethyl butyrate, (-)-lactic acid ethyl ester, and butyric acid ethyl ester were insufficiently synthesised, which would disrupt the flavour balance of the pit mud [8]. In pit C, the key compounds were mainly characteristic aroma esters, including butyric acid ethyl ester (18.56 mg/kg), (-)-lactic acid ethyl ester (105.68 mg/kg), hexanoic acid ethyl ester (19.76 mg/kg), and octanoic acid ethyl ester (27.32 mg/kg), with a balanced ratio of various ester and acid substances, and no abnormal metabolic product accumulation. This is also the core reason why the flavour quality of pit C is superior to that of pit A. In summary, the abnormal accumulation of acids such as heptanoic acid, octanoic acid, and heptanoic acid ethyl ester, as well as the insufficient synthesis of core aroma esters such as ethyl butyrate, (-)-lactic acid ethyl ester, and butyric acid ethyl ester, together constitute the key compounds for the off-flavours in pit A.

### 3.2. The Microbial Community Structure Related to Key Off-Flavours and the Differences in Microorganisms

**Figure 3:**
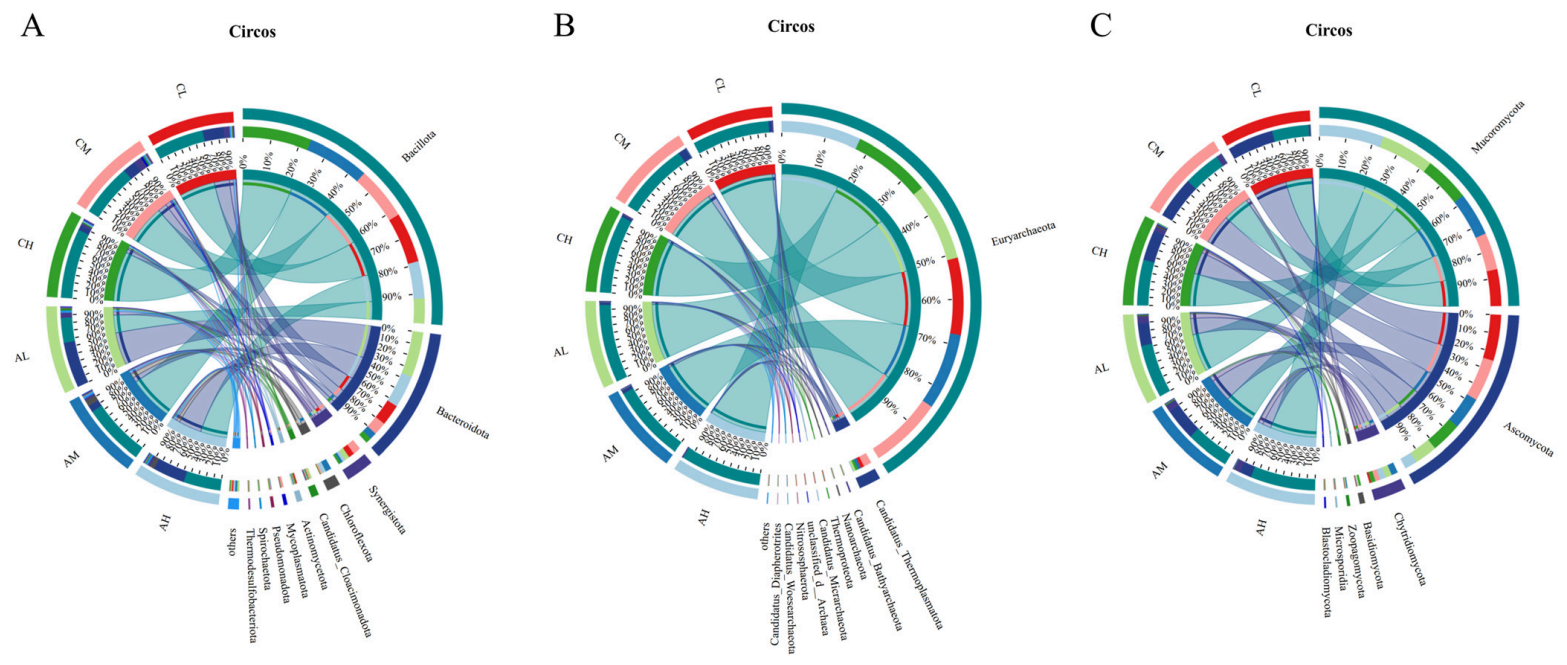
Microbial community composition at different pit levels ((**A**): Bacteria; (**B**): Archaea; (**C**): Fungi).

Metagenomic sequencing was employed to investigate microbial community composition in pit mud samples collected from different spatial locations within cellars of varying quality. By comparing results with the NR database, Figure 3 presents species annotation outcomes for both cellar groups, illustrating composition at phylum and genus levels across bacterial, archaeal, and fungal communities. Microbial communities with relative abundances exceeding 1% were defined as dominant microorganisms. The results indicate that in both pit A and pit C, at the bacterial phylum level (Figure 3A), Bacillota (51.74–39.64%), Bacteroidota (26.12–11.45%), Synergistota (4.55–3.15%), Chloroflexota (3.44–0.37%), and Candidatus_Thermoplasmatota (1.56–1.02%) were dominant. Bacillota was the absolutely dominant phylum across all samples. Within pit A, its abundance initially increased, then decreased with increasing pit depth, whereas within pit C, it exhibited a decreasing trend with increasing pit depth. Bacteroidota, as key drivers of the microbial carbon cycle in pit sludge, metabolise sugars to produce small-molecule acids. Their relative abundance exhibited a marked enrichment trend in pit sludge with declining mass [9]. At the phylum level (Figure 3B), Euryarchaeota (25.66–22.42%) and Candidatus Thermoplasmatota (1.56–1.02%) dominated; Euryarchaeota are important methanogenic microorganisms in pit mud. They decompose and ferment the organic acids (such as acetic acid, hydrogen gas, etc.) produced during the fermentation process, converting them into methane to maintain the anaerobic environment of the pit mud. In pit A, the trend is a decline as the depth of the pit pool increases. In pit C, it is the opposite [10]. Candidatus_Thermoplasmatota is regarded as a pivotal ‘mutualistic partner’ and ‘environmental responder’, indirectly supporting flavour compound synthesis networks—represented by hexanoic acid—by participating in organic acid metabolism and maintaining system stability [11]. At the fungal phylum level (Figure 3C), Mucoromycota (66.03–47.00%), Ascomycota (47.37–24.56%), and Chytridiomycota (77.43–33.82%) dominate. Mucoromycota were primarily active in the surface layer of the pit sludge and within the pit walls, while Ascomycota participated in processes such as starch degradation and organic acid metabolism. In the two groups of pit mud samples, the compositions of the dominant bacterial phyla and the dominant fungal phyla were relatively similar. However, in pit C, the relative abundances of Bacillota, Euryarchaeota, Synergistota, Caustatus_Thermoplasmatota, Actinomycetota and Ascomycota were higher than those in pit A. The synthesis of flavour compounds is closely related to microorganisms. The soil of pit A is more inclined to enrich aerobic or facultative anaerobic microorganisms, thereby to some extent suppressing the absolute dominance of anaerobic functional groups such as methanogens and caproic acid bacteria, resulting in an unpleasant odour in the soil of pit A. Pit C has enriched strictly anaerobic microorganisms such as Euryarchaeota and Synergistota, as well as acidophilic or acid-tolerant microorganisms such as Bacillota (many of which are acid-producing bacteria) and Ascomycota (fungi), with efficient and coordinated synthesis and esterification capabilities, which is conducive to the synthesis of a series of medium- and short-chain fatty acid ethyl esters and complex esters.

**Figure 4:**
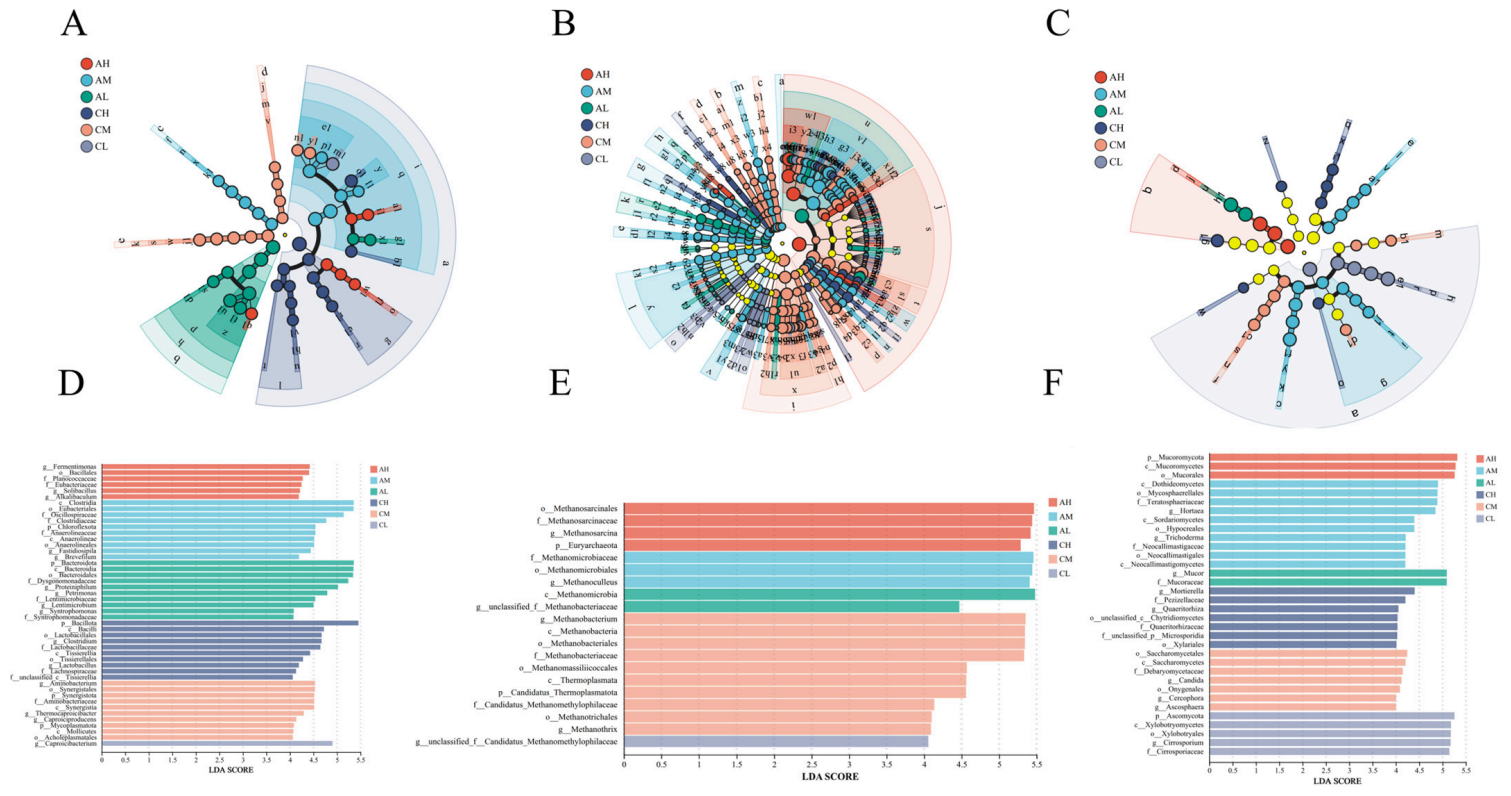
Microbial community composition differences in pit sludge from different pit muds ((**A**,**D**): Bacteria; (**B**,**E**): Archaea; (**C**,**F**): Fungi).

At the genus level, the two fermentation pits yielded 3534 bacterial genera, 257 archaeal genera, and 69 fungal genera. Among these, *Caproibacterium* (19.76–2.41%), *Proteiniphilum* (23.79–2.38%), *Petrimonas* (14.61–1.52%), *Clostridium* (11.30–1.81%), *Aminobacterium* (9.20–2.44%), and *Syntrophomonas* (3.68–1.28%) constituted the dominant bacterial genera (Figure 4D); *Methanosarcina* (71.24–7.33%), *Methanobacterium* (57.80–2.54%), and *Methanoculleus* (58.96–2.05%) were the dominant archaeal genera (Figure 4E); *Mucor* (46.66–25.58%), *Cirrosporium* (42.60–9.54%), *Rhizopus* (42.43–4.69%), *Hortaea* (18.17–5.78%), *Mortierella* (7.43–1.82%), *Rhizophagus* (4.73–0.85%) constituted the dominant fungal genera (Figure 4F). Among these, *Caproicibacterium* serves as the core microorganism responsible for generating the primary aroma. Its presence and activity represent crucial biomarkers for assessing pit mud health and predicting baijiu quality [12]. *Clostridium* constitutes one of the most critical core functional microbial communities, particularly renowned for its hexanoic acid production capability, which directly determines the characteristic flavour profile of Strong-Flavour Baijiu [13]. *Aminobacterium* primarily decomposes amino acids generated from the initial degradation of proteins within the pit mud, yielding primary metabolites such as acetic acid and hydrogen. *Methanobacterium* does not directly contribute flavour but thermodynamically ‘drives’ and ‘ensures’ the efficient synthesis of flavour precursors by the core functional community centred on hexanoate-producing bacteria by maintaining extremely low hydrogen partial pressure and a hyper-anaerobic environment [14]. *Cirrosporium* and *Mortierella* exhibit higher relative abundances in pit C compared to pit A; the physical and chemical environment of pit C is actively screening and enriching a stable symbiotic network centred on *Caproicibacterium* and *Clostridium*, with the participation of efficient co-collaborators such as Preparative Bacteria (e.g., *Aminobacterium*) and Terminal Consumers (e.g., *Methanosarcina*), thereby ensuring the efficient synthesis of flavour substances. *Proteiniphilum* catabolises proteins to produce propionic acid and acetic acid; *Petrimonas* degrades organic matter, primarily yielding acetic and propionic acids; *Syntrophomonas* engages in mutualistic interactions with methanogens, cooperatively degrading butyric acid and long-chain fatty acids, supplying products (e.g., H_2_, acetic acid) to methanogens; *Methanosarcina* and *Methanoculleus*, whose metabolism consumes acetate and hydrogen to maintain anaerobic conditions, safeguard acid-producing bacterial activity and serve as key members at the end of the carbon cycle [15]. *Mucor* may participate in preliminary material decomposition but exhibits low abundance in the core aroma-producing zone (pit bottom sludge). *Rhizopus* may contribute to the saccharification of starchy substrates, but is not a core anaerobic fermenter in pit sludge. *Hortaea* and *Rhizophagus* demonstrate higher relative abundances in pit A compared to pit C. The abundances of key bacterial groups such as acid-producing bacteria have decreased significantly, and the inter-relationship among them has been weakened. The microbial community mainly focuses on participating in the decomposition of raw materials in the early stage and the synthesis of non-dyke mud flavour substances. This might be one of the reasons for the obvious off-flavours in pit A.

To further investigate the microbial characteristics of pit mud samples of varying quality and assess their key functions, LEfSe analysis and the Wilcoxon rank-sum test were employed to screen for differentially abundant species across pit mud samples of differing quality and strata. Microorganisms with LDA values exceeding four were defined as differentially abundant. Results are presented in Figure 4, where inter-group analysis detected 15 bacterial genera, six archaeal genera, and nine fungal genera across six samples. Specifically, bacterial and archaeal genera specifically enriched in the upper layer of pit A included *Fermentimonas*, *Solibacillus*, *Alkalibaculum*, and *Methanosarcina*; the middle layer was characterised by *Fastidiosipila*, *Brevefilum*, *Methanoculleus*, *Hortaea*, and *Trichoderma*; while the bottom layer specifically contained *Proteiniphilum*, *Petrimonas*, *Lentimicrobium*, *Syntrophomonas*, *unclassified_f__Methanobacteriaceae*, and *Mucor*. The microorganisms enriched in pit A are more likely to be aerobic or facultative anaerobic decomposers rather than strictly anaerobic synthesisers. They (such as *Fastidiosipila*, *Hortaea*, *Trichoderma*, *Mucor*, etc.) are more related to the primary decomposition process of large molecules such as starch and protein, and have failed to form a tight and continuous vertical functional network like that in pit C, which is oriented towards the final synthesis of butyric acid. In contrast, the upper layer of pit C harboured *Clostridium*, *Lactobacillus*, *Mortierella*, and *Quaeritorhiza* as specific bacterial and fungal genera; the middle layer showed specific enrichment of *Aminobacterium*, *Thermocaproicibacter*, *Caproiciproducens*, *Methanobacterium*, *Methanothrix*, *Candida*, *Cercophora*, and *Ascosphaera*; the bottom layer primarily yielded *Caproicibacterium*, *unclassified_f__Candidatus_Methanomethylophilaceae*, and *Cirrosporium*. Notably, the marker microorganisms enriched in pit C, such as *Caproicibacterium*, *Methanobacterium*, and *Cirrosporium*, have been reported to be significantly associated with the production of key volatile compounds, including acetic acid, ethyl acetate, and ethyl caproate [16]. These biomarkers may influence the fermentation function and flavour compound synthesis of pit sludge by participating in multiple metabolic pathways. Conversely, the dominant marker microorganisms in pit A are primarily associated with the degradation processes of macromolecules such as starch and protein, suggesting their metabolic functions are more oriented towards primary substrate degradation. The stable anaerobic environment of the cellar has shaped a coherent and efficient microbial combination for flavour synthesis from the top to the bottom. The bottom layer is the core aroma-producing area, where the specific rich *Caproicibacterium* producer of caproic acid is highly dependent on strict anaerobic and low hydrogen partial pressure conditions, which is consistent with the stable high-water and low-redox-potential conditions at the cellar bottom [17]. The middle layer is the key mutualistic metabolic area, where *Aminobacterium* (producing acetic acid/hydrogen), *Thermocaproicibacter* (producing caproic acid), and key hydrogen-dependent methanogen *Methanobacterium* and acetic acid-dependent methanogen *Methanothrix* are enriched. The upper layer then presents Clostridium (acid-producing bacteria) and Lactobacillus, which may undertake the functions of acid production in the early stage of fermentation and creation of an anaerobic microenvironment. This microbial succession from top to bottom and spatial division form a complete and efficient flavour substance (especially caproic acid and its esters) synthesis pipeline. The participation of fungi such as *Cirrosporium* may further assist this metabolic network [18]. The layered distribution pattern of different microorganisms clearly reveals that there exists a ‘flavor-producing microbial community’ with an orderly spatial structure and coordinated metabolic functions in pit C. Key indicators (such as *Caproicibacterium* and *Methanobacterium*) are present throughout. However, the community in pit A exhibits fragmented functional characteristics, with the dominant bacterial groups favouring substrate decomposition and lacking a core hub and collaborative driving force for efficient synthesis of flavour substances. From the perspective of the spatial distribution of biomarkers, this further confirms that the structural differences in microbial communities are the fundamental cause leading to the differentiation of fermentation functions and final quality in the pit.

### 3.3. Predicting the Microbial Populations Related to the Off-Flavours of Pit Mud

**Figure 5:**
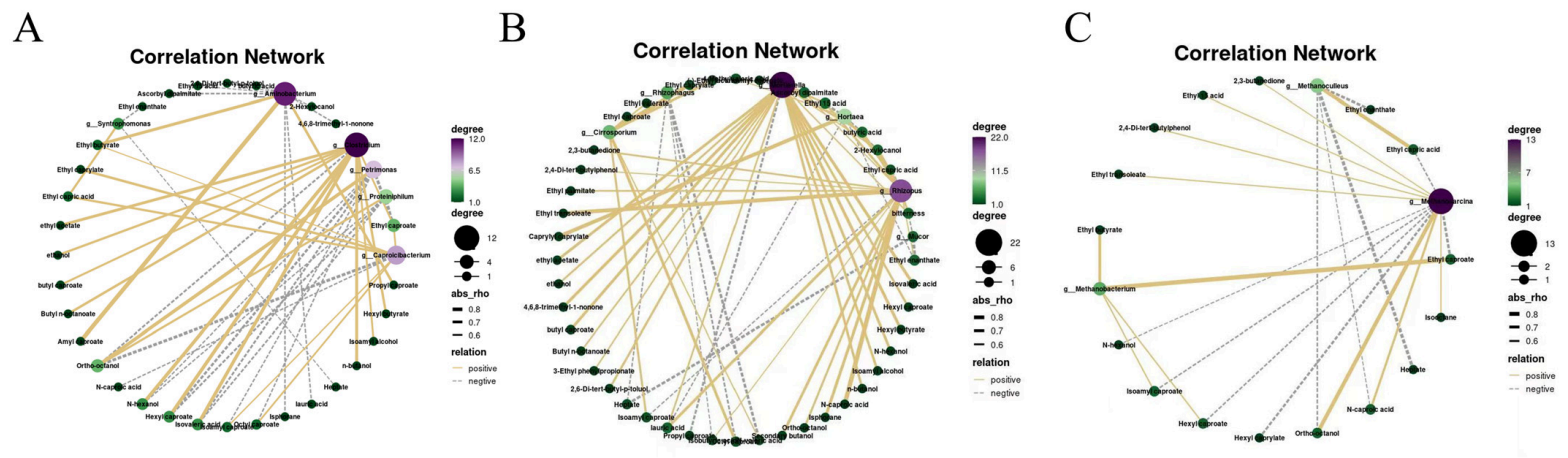
Network diagram of microbial communities and volatile metabolites in pit mud ((**A**): Bacteria; (**B**): Archaea; (**C**): Fungi).

To identify the microorganisms associated with the off-flavours of pit mud, Spearman correlation analysis was used to reveal the correlations between the dominant bacteria, archaea, and fungal microorganisms and 14 key flavour compounds (Figure 5). 

The results indicate that *Methanobacterium* exhibits positive correlations with esters, including ethyl butyrate, ethyl caproate, isoamyl caproate, and hexyl caproate, focusing on short-chain ester metabolism and contributing to foundational ester aromas [19]. *Caproiciproducen* targeted characteristic esters, exhibiting positive correlations with key liquor flavour esters including ethyl acetate, ethanol, n-butanol, isoamyl alcohol, ethyl caproate, butyl caproate, and butyl n-octanoate, thereby further enhancing the complexity and richness of the pit mud’s ester aroma [20]. *Aminobacterium* centres on alcohol metabolism, exhibiting positive correlations with butyric acid, lauric acid, and ethyl 15-acid, participating in the decomposition and conversion of amino acids within pit mud [21]. *Cirrosporium* focuses on auxiliary foundational flavouring, positively correlated with ethyl valerate, ethyl caproate, (-)-ethyl lactate, ethyl caprylate, isoamyl caproate, octyl caproate, and N-valeric acid, participating in the metabolic processes of fundamental aroma compounds to establish the foundational framework for the overall flavour profile. *Rhizopus* exhibits positive correlations with ethyl enanthate, butyric acid, N-caproic acid, and bitterness, which significantly enrich flavour complexity and uniqueness through metabolic activities [22]. These compounds exhibit positive correlations with most esters and demonstrate higher relative abundance in pit C compared to pit A. *Proteiniphilum*, focusing on flavour balance regulation, exhibits negative correlations with ethyl caproate, N-hexanol, hexyl caproate, and isovaleric acid. By modulating these compounds’ levels, it maintains the overall harmonious character of the pit mud’s flavour profile [23]. *Petrimonas* focuses on ester flavour regulation, exhibiting negative correlations with ethyl caproate, n-hexanol, isoamyl caproate, hexyl caproate, isovaleric acid, and octyl caproate. *Rhizopus*-associated flavour compounds cover a broader spectrum, showing positive correlations with butyric acid, lauric acid, n-caproic acid, and bitterness. Reports indicate this species exhibits high abundance during early fermentation stages [24]. *Methanoculleus* correlates positively with ethyl enanthate, ortho-octanol, N-caproic acid, and heptate, participating in ketone and acid flavour compound production [25]. *Hortaea* centres its activity on acid metabolism, exhibiting negative correlations with ethyl enanthate, 2-hexylocanol, butyric acid, lauric acid, ethyl 15-acid, and ascorbyl dipalmitate. It contributes to the formation of acidic aroma characteristics within the flavour profile and assists in regulating the acid–sweet balance. Its relative abundance is greater in fermentation pit A than in pit C.

The differences in microbial community structure are the core reason for the differentiation of pit mud flavour characteristics and the generation of off-flavours: On one hand, there are significant differences in the types and relative abundances of dominant functional microorganisms between pit A and pit C. The enrichment of ester-smelling synthesising microorganisms (such as *Methanobacterium*, *Caproiciproducens*, *Rhizopus*, etc.) in pit C promotes the efficient synthesis of beneficial flavour substances such as short-chain esters and alcohols, resulting in a rich and harmonious flavour profile. While in pit A, the proportion of regulatory microorganisms (such as *Proteiniphilum*, *Petrimonas*, *Hortaea*, etc.) is higher, although they can maintain local flavour balance, excessive enrichment may lead to insufficient synthesis of ester-smelling substances, disrupting the flavour ratio of ‘ester–alcohol–acid’. On the other hand, when the microbial community is imbalanced, abnormal proliferation or absence of specific microorganisms can trigger metabolic disorders: if acid metabolism-related microorganisms (such as *Hortaea*) are overly enriched, it may lead to excessive accumulation of organic acid substances, resulting in sour off-flavours; if the abundance of ester synthesising microorganisms (such as *Caproiciproducens*) is too low, it will result in weak ester-smelling substances, making the pit mud flavour monotonous or even introducing off-flavours. In summary, the composition and functional differentiation of the pit mud microbial community directly determine the types, contents, and proportion balance of volatile flavour compounds. The imbalanced state of the microbial community is the fundamental cause of the flavour differences in pit mud and a key factor in the generation of off-flavours.

### 3.4. Analysis and Prediction of Microbial Community Functions in Pit Mud

**Figure 6:**
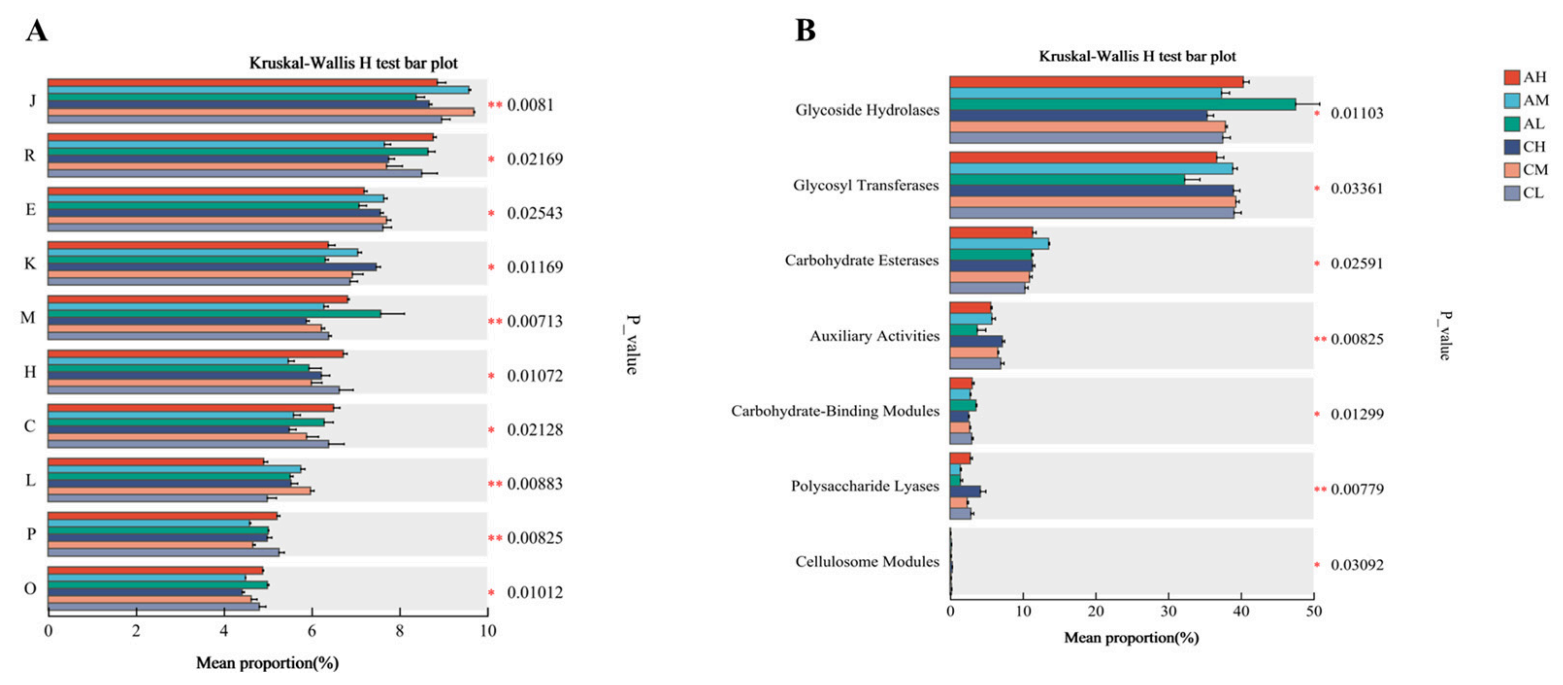
Functional diversity of microbial communities in normal and off-flavoured pit mud across different layers, based on functional annotation using EggNOG (**A**) and Cazy (**B**) (*: *p* < 0.05, **: *p* < 0.01).

This study employed Megahit and Prodigal for metagenomic assembly and functional prediction, respectively. A total of 1,043,800 unique genes were obtained across six samples. These genes underwent functional annotation and matching via eggNOG, CAZy, and KEGG databases (e-value ≤ 10). These genes were clustered into 21 eggNOG categories (Figure 6A). The top three categories in pit A and pit C are J (translation, ribosomal structure and biosynthesis), R (general function prediction only), and E (amino acid transport and metabolism). This reflects the common requirements of microbial communities for maintaining basic life activities and core metabolism. The second group is K: transcription; M: cell wall/membrane/envelope biogenesis; H: coenzyme transport and metabolism; C: energy production and conversion; L: replication, recombination and repair; P: inorganic ion transport and metabolism; and O: posttranslational modification, protein turnover, chaperones. The two groups of biomes exhibit different strategies and focuses, which are closely related to their different physical and chemical environments and community niches. In pit A, the most abundant eggNOG families are R, M, C and O. The microbial community in this pit allocates more resources to environmental adaptation, stress response and cell structure maintenance. In pit C, the most abundant families are J, E, K, H, L and P. This functional profile indicates that the microbial community in pit C is in a state of active metabolism and vigorous growth and reproduction. The abundant amino acid metabolism and coenzyme metabolism genes support the metabolic network for efficient synthesis of flavour precursor substances (such as acetic acid and caproic acid) [26]; this functional difference provides an intrinsic mechanistic basis for explaining the final quality differentiation between the two groups of pits.

According to the CAZy database annotations, as shown in Figure 6B, these genes across the six samples were classified into six categories: Glycoside Hydrolases, Glycosyltransferases, Carbohydrate Esterases, Auxiliary Activities, Carbohydrate-Binding Modules, Polysaccharide Lyases, and Cellulosome Modules. As anticipated, the most abundant CAZy family in Sample A was GH, followed by CE and GBM. The GH family enzymes mainly catalyse the hydrolysis of glycosidic bonds, directly participating in the degradation of complex polysaccharides such as starch and cellulose, providing substrates for glycolysis and pyruvate metabolism [27]. This indicates that the microbial community in pit A may focus its metabolic centre on breaking down large-molecule carbohydrates into usable monosaccharides or oligosaccharides, which belongs to the primary stage of raw material utilisation. The pathways significantly enriched in pit C include GT, AA, PL, and CM. The GT family is involved in the synthesis and modification of oligosaccharides and polysaccharides, supporting a more advanced and synthesis-oriented metabolic system [21].

**Figure 7:**
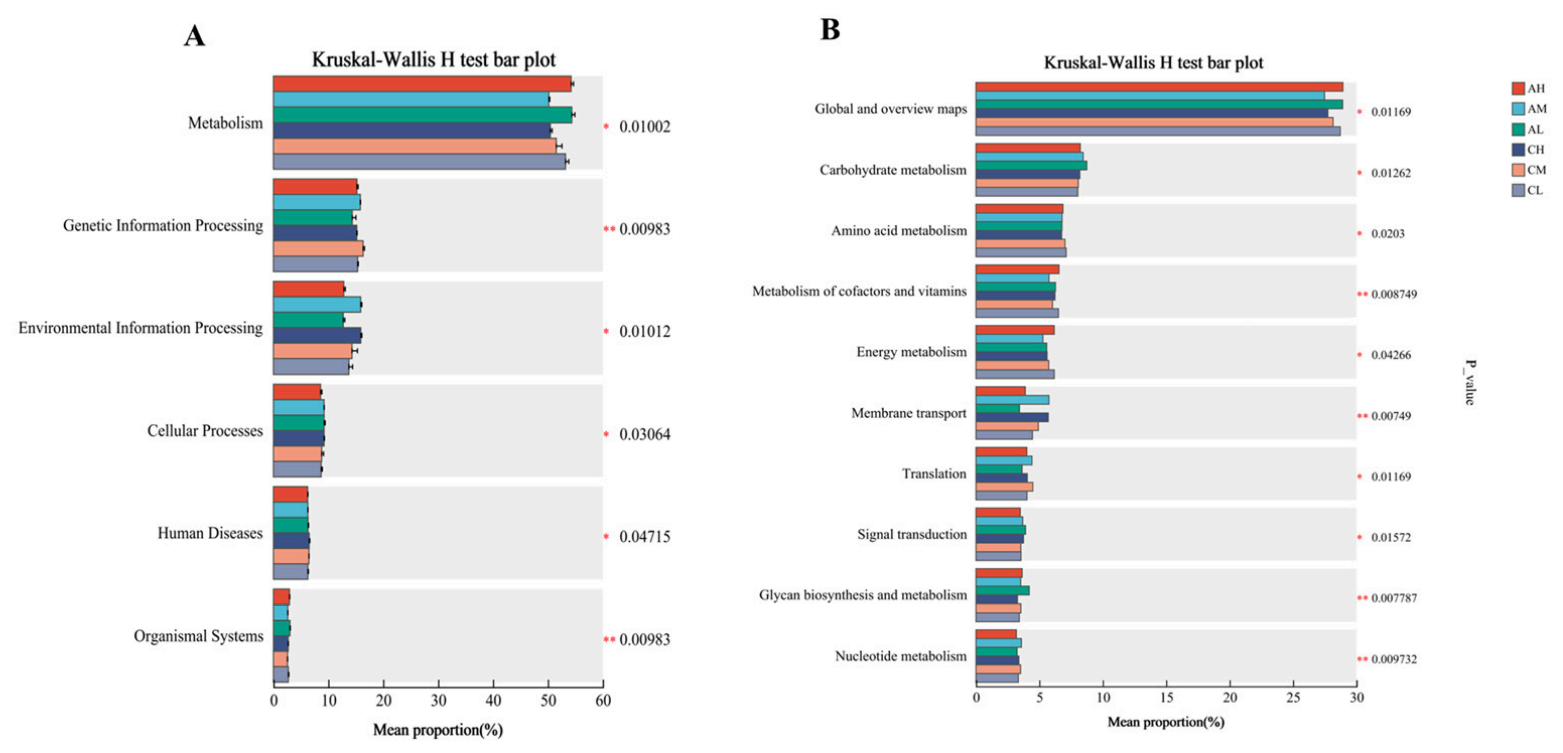
Functional diversity of microbial communities in different layers of metabolic pathways associated with the formation of key off-flavoured pit mud based on KEGG functional annotation. ((**A**): Based on KEGG Level 1 functional annotation; (**B**): based on KEGG Level 2 functional annotation; (*: *p* < 0.05, **: *p* < 0.01)).

The KEGG pathways involved in each microbial gene during the fermentation process were analysed. The results showed that the functional pathways were divided into three subcategories (Figure 7). In Level 1, metabolism (ME) was the most annotated pathway, followed by genetic information processing (GIP), environmental information processing (EIP), cell processing processes, human diseases, and biological systems. This highlights that the core activities of the pit mud microbial community are the metabolism and energy utilisation of substances, and it possesses corresponding environmental adaptation and genetic regulation capabilities. Further focusing on the metabolic pathways at Level 2, global and overview maps, carbohydrate metabolism, and amino acid metabolism were identified as the core pathways, forming the basic metabolic framework of the pit mud fermentation. At the more detailed Level 3, a series of pathways closely related to the synthesis of flavour substances were significantly enriched, among which the central carbon metabolism pathway was the most prominent, followed by the biosynthesis of secondary metabolites, microbial metabolism under different environments, cofactor biosynthesis, amino acid biosynthesis, and carbon metabolism, etc. The enrichment of these pathways is conducive to the formation of various flavour compounds [16]. The KEGG pathway analysis from the systems biology perspective clarifies that the active and balanced central metabolism and synthetic metabolism network in the pit mud is the fundamental reason for its efficient production of rich and coordinated flavour compounds [6]. Although pit A has a similar metabolic pathway framework to pit C, its activity and efficiency in specific pathways (especially synthetic metabolism pathways) may be inhibited due to the imbalance in the physical and chemical environment and the disorder in the community structure, resulting in insufficient flavour synthesis ability. This further provides a mechanistic explanation for the quality differentiation of cellars from the perspective of the activity and distribution of metabolic pathways.

### 3.5. Metabolic Pathways Associated with the Formation of Key Off-Flavours

**Figure 8:**
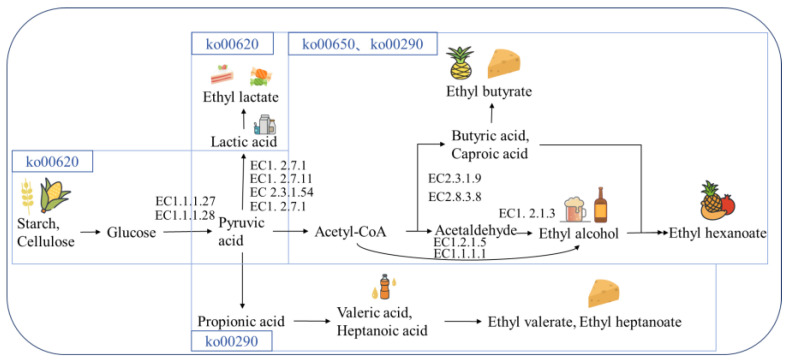
Metabolic pathways associated with the formation of key off-flavoured compounds.

The metabolic pathways associated with the formation of key off-flavour compounds are illustrated in Figure 8 Within the pit sludge metabolic network, the degradation of starch, cellulose, and other substances yields glucose. Following catalytic utilisation, glucose is converted into pyruvate, which serves as a node through which multiple compounds are formed via distinct metabolic pathways. Regarding lactic acid metabolism, pyruvate serves as a hydrogen acceptor for NADH (reduced coenzyme I). Through the reversible catalysis of two enzyme classes—EC 1.1.1.27 (L-lactate dehydrogenase) and EC 1.1.99.7 (lactate–malate transhydrogenase)—L-lactic acid is generated. Lactic acid further undergoes esterification with ethanol to form ethyl lactate [28]. The enzymes involved in lactate production generally have a relatively high abundance in the A fermentation tank. This may be due to the relatively high pH environment, which is more conducive to the activity of related microorganisms (such as certain lactic acid bacteria), resulting in excessive lactate production. Although lactic acid ethyl ester is one of the flavour components of Baijiu, the excessive accumulation of the precursor lactate may interfere with the overall ester balance. The synthesis of acids such as butyric and hexanoic acids likewise originates from pyruvate. Pyruvate is catalysed by EC 1.2.7.1 (pyruvate synthase, i.e., 2-oxo acid oxidoreductase) and EC 2.3.1.54 (formate C-acetyltransferase) to form acetyl-CoA. Two molecules of acetyl-CoA condense under the catalysis of EC 2.3.1.9 (acetyl-CoA acetyltransferase) to form acetyl-CoA, which subsequently combines with acetyl-CoA. Through extended metabolic pathways, this complex progressively forms butyryl-CoA and hexanoyl-CoA. Finally, under the action of coenzyme A transferases (e.g., EC 2.8.3.8), butyryl-CoA and hexanoyl-CoA are converted into butyric acid and hexanoic acid, respectively. These key enzymes also exhibit high relative abundance in Tank A. Butyric acid and hexanoic acid can undergo further esterification with ethanol to yield butyl ethanoate and hexyl ethanoate, respectively [29].

The accumulation of valeric acid (detected at 2.19 mg/kg) is closely associated with an increase in the relative abundance of the valine, leucine, and isoleucine degradation pathway (enrichment factor FC: 7.57), which promotes the synthetic metabolism of valeric acid. This degradation pathway encompasses key enzyme systems for short-chain fatty acid synthesis: first, branched-chain amino acid aminotransferase (EC 2.6.1.42) catalyses the conversion of the aforementioned three amino acids into their corresponding α-keto acids; subsequently, α-keto acids are converted into acylated short-chain fatty acids via α-ketoacid decarboxylase (EC 1.2.4.4) and dihydrosuccinyl transferase (EC 2.3.1.168); finally, free short-chain fatty acids are released through catalysis by chitinase (EC 3.2.1.14).

The heptanoic acid content in pit A was higher than that in pit C. Hexanoic acid in the pit sludge is primarily synthesised via the microbial reverse β-oxidation pathway. Notably, the metabolic pathways of hexanoate-producing bacteria are significantly influenced by environmental pH: within the normal range of pH 5–7, metabolic flux favours hexanoate synthesis; however, when pH falls below 5, the growth and metabolism of hexanoate bacteria are inhibited, potentially leading to sludge ageing and odour production.

### 3.6. The Effect of the Nutritional Components of the Pit Mud on Microorganisms

**Figure 9:**
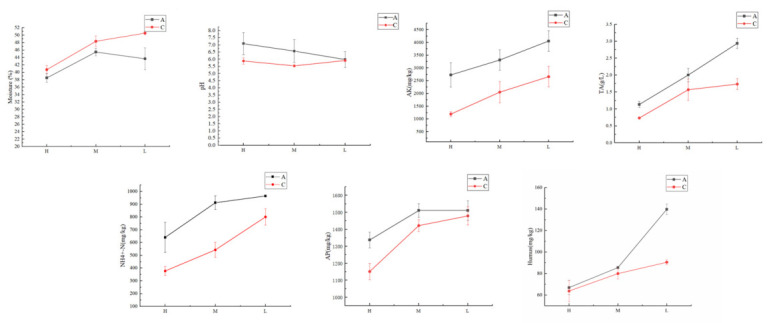
Nutritional elements of normal and off-flavoured pit mud.

The distribution of microbial communities in pit A and pit C in the vertical space is not random; it is the result of the internal nutrient components selectively influencing the microorganisms in a systematic manner. This study compared moisture content, pH, ammonium nitrogen, available phosphorus, readily available potassium, and organic matter content across different layers of cellar sludge from two distinct cellar types. The results are presented in Figure 9.

Significant differences in moisture content were not observed between the pits. In pit A, the moisture contents of the upper, middle and lower layers of the soil are 38.49%, 45.46% and 43.65%, respectively. Water generated by microbial community metabolism gradually accumulates during fermentation and settles at the pit bottom. In pit C, sludge moisture content increased with pit depth; in pit A, it initially rose, then declined with increasing depth. Notably, the moisture content in pit C consistently exceeded that in pit A. High water content indicates a strict anaerobic environment and high microbial metabolic activity, which is consistent with the composition of the microbial community in pit C [3]. pH constitutes a critical factor influencing pit sludge quality, directly or indirectly affecting microbial growth and reproduction. Significant pH variations were observed among sludge samples from pits of differing quality; as shown in Figure 9, the pH values of the upper, middle and lower layers of pit A’s pit mud were approximately 7.09, 6.57 and 5.98, respectively, while the pH of pit C remained consistently at around 5.77. The average pH of pit A was significantly higher than that of pit C (*p* < 0.05). During the solid-state fermentation process, the organic acids produced by the metabolic activities of functional microorganisms (especially acid-producing bacteria) cause the pit mud environment to become acidic [30]. The lower pH and higher total acid content of pit C indicate that the microbial community of acetic acid bacteria and others is more active. On the contrary, the higher pH of pit A may inhibit the growth and metabolism of beneficial microorganisms such as acetic acid bacteria, which may be one of the important reasons for the deterioration in quality in pit A [31]. Ammonium nitrogen serves as the primary nitrogen source for microbial growth and reproduction in sludge; the ammonium nitrogen content in the upper, middle and lower layers of pit A was approximately 640.94 mg/kg, 912.09 mg/kg and 964.86 mg/kg, respectively. Its content was consistently higher than that of pit C (*p* < 0.05). Ammonium nitrogen content exhibited an increasing trend with greater pit depth, with ammonium nitrogen levels consistently higher in pit A sludge than in pit C sludge. This disparity reflects relatively lower nitrogen utilisation efficiency and weaker metabolic activity among pit A microorganisms, leading to gradual accumulation of unassimilated nitrogen within the system. This accumulation may further compromise enzyme activity and the coordination of microbial metabolic pathways, causing pit A to have excessive nutrients [32]. Available phosphorus (primarily comprising water-soluble phosphorus and adsorbed phosphorus) and readily available potassium (water-soluble potassium) are essential mineral elements for microbial growth, directly participating in energy transfer, cell structure formation, and various enzymatic reactions. Organic matter, meanwhile, provides crucial carbon sources and energy for microorganisms, sustaining the metabolic foundation of the pit sludge ecosystem [33]. Analytical data indicate that pit A slurry exhibits higher levels of available phosphorus, readily available potassium, and organic matter compared to pit C slurry (Figure 2). Although elevated nutrient levels are typically regarded as beneficial for microbial growth, observational evidence suggests that nutrient excess may disrupt optimal growth thresholds, inhibit the activity of certain functional microbial communities, and interfere with normal microbial community succession and functional expression. This, in turn, compromises the overall structure and fermentation stability of the slurry. Nutritional imbalance may disrupt microbial metabolic pathways, diminishing the performance of pit sludge during prolonged fermentation. Therefore, maintaining an appropriate nutritional dynamic balance is crucial for ensuring the healthy succession and high-quality fermentation of the microbial community in the pit mud.

## 4. Conclusions

We analysed the metabolites, microbial community and nutritional elements of pit mud from cellars of varying quality producing Strong-Flavour Baijiu. The data show that the abnormal accumulation of acid substances such as heptanoic acid and caproic acid and the deficiency in the synthesis of key esters disrupt the flavour balance of the pit mud, causing it to have a strong sour and foul smell. The off-flavours in the pit mud mainly originate from acid-tolerant and aerobic microorganisms such as *Fermentimonas*, *Solibacillus*, *Alkalibaculum*, *Methanosarcina*, *Fastidiosipila*, *Brevefilum*, *Methanoculleus*, *Hortaea*, *Trichoderma*, *Proteiniphilum*, *Petrimonas*, *Lentimicrobium*, *Syntrophomonas*, *unclassified_f__Methanobacteriaceae* and *Mucor*. The starch and cellulose degradation pathways, the biosynthesis pathways of valine, leucine and isoleucine, the pyruvate metabolism pathway, the butyric acid metabolism pathway, and the analysis of nutrient elements in the pit mud all indicate that nutritional imbalance may cause disorders in microbial metabolic pathways, thereby reducing the performance of the pit mud during the fermentation process. In summary, this study first established the complete mechanism chain of ‘nutrient excess–microbial community disruption–activation of off-flavour metabolic pathways’. It clarified that nutrient excess (nitrogen, phosphorus, and potassium enrichment) is the initial trigger for silage degradation, microbial community structural imbalance is the core driver, and the activation of specific metabolic pathways is the direct cause. Based on this, targeted measures such as optimising raw material ratios (avoiding excessively high-protein and high-phosphorus inputs), regulating pit mud pH to the optimal range of 5–7, and supplementing functional bacterial agents like Caproicibacterium can achieve precise prevention and control of pit mud off-flavours. This research provides theoretical underpinnings and technical guidance for enhancing pit mud quality and controlling off-flavours in Strong-Flavour Baijiu, while also establishing a novel research paradigm for microbial regulation in fermented food production.

## Data Availability

The original contributions presented in this study are included in the article/Supplementary Materials. Further inquiries can be directed to the corresponding author.

## Supplementary Materials

The following supporting information can be downloaded at https://www.mdpi.com/article/10.3390/foods15152731/s1, Figure S1: Sequencing depth of normal and off-flavor pit mud.

## References

[1] Dong W., Guo R., Liu M., Shen C., Sun X., Zhao M., Sun J., Li H., Zheng F., Huang M. (2019). Characterization of key odorants causing the roasted and mud-like aromas in strong-aroma types of base Baijiu. Food Res. Int..

[2] Liu M., Tang Y., Zhao K., Liu Y., Guo X.-J., Tian X.-H., Ren D.-Q., Yao W.-C. (2019). Contrasting bacterial community structure in artificial pit mud-starter cultures of different qualities: A complex biological mixture for Chinese strong-flavor Baijiu production. 3 Biotech..

[3] Gong Y., Ma N., Tang H. (2022). Analysis of microbial community diversity and physicochemical factors in pit mud of different ages based on high-throughput sequencing. Can. J. Microbiol..

[4] Li J., Ding Z., Dong W., Li W., Wu Y., Zhu L., Ma H., Sun B., Li X. (2024). Analysis of differences in microorganisms and aroma profiles between normal and off-flavor pit mud in Chinese strong-flavor Baijiu. J. Biosci. Bioeng..

[5] Han B., Gong H., Ren X., Tian S., Wang Y., Zhang S., Zhang J., Luo J. (2024). Analysis of the differences in physicochemical properties, volatile compounds, and microbial community structure of pit mud in different time spaces. PeerJ.

[6] Gong J., Zuo Q., Wu Z., Zhao C., Wei J., Huang Y. (2024). Unraveling the core microorganisms and metabolic pathways related to off-flavor compounds formation during Jiang-flavor Baijiu fermentation. Food Chem. X.

[7] Gao J. (2025). Research on the Flavor Contribution and Homeostatic Mechanism of Microbial Communities in the Mud Pit of Strong-Aroma Chinese Baijiu. Ph.D. Thesis.

[8] Zeng X., Liu L., Li D., Li T., Wei C., Li D., Yan Y., Cao W. (2025). Insight into the succession pattern of the microbial community of fermented grains and driving forces during pit fermentation of Maotai-flavor baijiu. Food Res. Int..

[9] Wang Y., Tian Y., Zhang K., Zhang S., Gao J., Tang H., Song C., Tang Y., Sun M., Zuo Y. (2025). Comparative Analysis of Microbial Community Diversity and Flavor Compounds in Distilled Liquor Mud Pits from Different Seasons and Levels and Their Correlation. Food Sci..

[10] Xiao Q., He P., Zhou R., Liu C., Yuan S., Zhao J. (2023). Comparative Analysis of Microbial Community Diversity and Physicochemical Factors in Distilled Liquor Mud from Different Ages and Locations of Strong Aroma Chinese Baijiu. Food Sci..

[11] Wang H., Gu Y., Zhou W., Zhao D., Qiao Z., Zheng J., Gao J., Chen X., Ren C., Xu Y. (2021). Adaptability of a Caproate-Producing Bacterium Contributes to Its Dominance in an Anaerobic Fermentation System. Appl. Environ. Microbiol..

[12] Sun H., Chai L., Fang G., Lu Z., Zhang X., Wang S., Shen C., Shi J., Xu Z. (2022). The Interactions between Clostridia and Synergistic Bacillus in Mud Pits and Their Effects on Growth and Short-Chain Fatty Acid Metabolism. Food Ferment. Ind..

[13] Zhao Q., Zhu H., Tong X., Bao G., Yang S., Wang S., Shen C., Li Y. (2023). Comparative genomic analyses reveal carbohydrates-rich environment adaptability of *Lentilactobacillus laojiaonis* sp. nov. IM3328. Food Biosci..

[14] Cheng W., Chen X., Xue X., Lan W., Zeng H., Li R., Pan T., Li N., Gong Z., Yang H. (2024). Comparison of the Correlations of Microbial Community and Volatile Compounds between Pit-Mud and Fermented Grains of Compound-Flavor Baijiu. Foods.

[15] Hong J., Huang H., Zhao D., Sun J., Huang M., Sun X., Sun B. (2023). Investigation on the key factors associated with flavor quality in northern strong aroma type of Baijiu by flavor matrix. Food Chem..

[16] Zeng Y., Zhong X., Chai L., Zhang X., Lu Z., Liu G., Tu T., Lu L., Zhang R., Yu H. (2025). Prokaryotic evolution shapes specialized communities in long term engineered pit mud ecosystem. npj Biofilms Microbiomes.

[17] Suo M., Liu L., Fan H., Li N., Pan H., Hrynsphan D., Tatsiana S., Robles-Iglesias R., Wang Z., Chen J. (2025). Advancements in chain elongation technology: Transforming lactic acid into caproic acid for sustainable biochemical production. Bioresour. Technol..

[18] Huang T., Wei J., Zhou S., Hao W., Liu X., Yu Z., Zhuang W., Zeng Z. (2025). Fungal community structure and succession of light-flavor Baijiu during fermentation process. LWT.

[19] He H., Yuan M., Cai Z., Cao R., Li A., Zhang Z. (2019). Design of Specific Primers for Methanococcus Genus and Its Preliminary Application in Quality Evaluation of Ancient Jiugeng Liquor Mud. Chin. Brew..

[20] Kang J., Huang X., Li R., Zhang Y., Chen X.-X., Han B.-Z. (2024). Deciphering the core microbes and their interactions in spontaneous Baijiu fermentation: A comprehensive review. Food Res. Int..

[21] Chen C., Yang H., Zhang K., Ye G., Luo H., Zou W. (2024). Revealing microbiota characteristics and predicting flavor-producing sub-communities in Nongxiangxing baijiu pit mud through metagenomic analysis and metabolic modeling. Food Res. Int..

[22] Zhou S., Chen B., Xu J., Yu Z., Dai Y., Li Z., Zhang K., Hou A. (2025). Changes in flavor compounds and microbial communities during Changle Sweet Rice Wine fermentation. LWT.

[23] Zeng X., Zou Y., Li D., Wei C., Li D., Yan Y., Lin Q. (2025). Systematically exploring and evaluating core fungal composition and their flavor profile in fermented grains of Jiang-flavor baijiu. Food Chem. X.

[24] Xie J., Hong J., Zhang C., Yuan X., Zhao Z., Zhao D., Wang S., Sun B., Ao R., Sun J. (2025). Machine learning-assisted identification of core flavor compounds and prediction of core microorganisms in fermentation grains and pit mud during the fermentation process of strong-flavor Baijiu. Food Chem..

[25] Liu Z., Fang C., Zhang X., Liu L., Mu J., Lin L., Zhang C. (2023). Research Progress on Physical-Chemical Factors, Microbial Communities, Sensory Quality and Cor-relation of Strong-Aroma Chinese Baijiu Mud Pits. Food Sci..

[26] Zhao Y., Tong C., Gu C., Xu Q., Yao W., Qian H., Cheng Y. (2025). Untargeted flavoromics and correlation analysis reveal microbial interactions driving flavor in LAB co-fermented Beita juice. Int. J. Food Microbiol..

[27] Liang Y.-P., Zhao Y.-L., Yin Z.-W., Gong X.-W., Han X.-L., Wen M.-L. (2025). Conserved Local Structural Motifs in Glycoside Hydrolase Families Facilitate the Discovery of Functional Enzymes. J. Agric. Food Chem..

[28] Mao F., Huang J., Zhou R., Zhang S., Qin H. (2024). The influence of artificial pit mud microbial community on the formation of flavor metabolites during the fermentation process of strong-flavor Chinese Baijiu. Food Sci..

[29] Zhang H., Xing X., Wang Y., Cui L., Wang X., Chang Q., Sun W., Xi X., Xue Z. (2024). Research Progress of Acetic Acid Bacteria in the Fermentation System of Strong-Aroma Chinese Baijiu. Food Sci..

[30] Shoubao Y., Yonglei J., Qi Z., Shunchang P., Cuie S. (2023). Bacterial diversity associated with volatile compound accumulation in pit mud of Chinese strong-flavor baijiu pit. AMB Express.

[31] Gao J., Qin J., Ye F., Ding F., Liu G., Li A., Ren C., Xu Y. (2022). Constructing simplified microbial consortia to improve the key flavour compounds during strong aroma-type Baijiu fermentation. Int. J. Food Microbiol..

[32] Wu C., Hu J., Xie D., Fan E., Yang J., You X., Cheng P., Huang W., Hu F., Wang D. (2024). Comparison of physicochemical parameters, microbial community composition and flavor substances during mechanical and traditional brewing process of Jiang-flavor baijiu. Food Sci. Biotechnol..

[33] Liu M., Tang Y., Guo X.-J., Zhao K., Tian X.-H., Liu Y., Yao W.-C., Deng B., Ren D.-Q., Zhang X.-P. (2017). Deep sequencing reveals high bacterial diversity and phylogenetic novelty in pit mud from Luzhou Laojiao cellars for Chinese strong-flavor Baijiu. Food Res. Int..

